# A Health Professional Mentorship Platform to Improve Equitable Access to Abortion: Development, Usability, and Content Evaluation

**DOI:** 10.2196/63364

**Published:** 2025-02-19

**Authors:** Abdul-Fatawu Abdulai, Cam Duong, Eleni Stroulia, Efrat Czerniak, Rachel Chiu, Aashay Mehta, Ken Koike, Wendy V Norman

**Affiliations:** 1 School of Nursing, University of British Columbia, Vancouver, BC, Canada; 2 Computing Science, Faculty of Science, University of Alberta, Edmonton, AB, Canada; 3 Cognitive Sciences, University of British Columbia, Vancouver, BC, Canada; 4 The Society of Obstetricians and Gynaecologists of Canada, Ottawa, ON, Canada; 5 Department of Family Practice, Faculty of Medicine, University of British Columbia, Vancouver, ON, Canada

**Keywords:** medication abortion, mifepristone, web-based platform, user-centered design, underserved populations, abortion, equitable, accessibility, open-access website, gender-affirming, user-centered, Canada, unwanted pregnancy, framework

## Abstract

**Background:**

Access to safe abortion care is a reproductive right for all individuals across Canada. Underserved populations are overrepresented among those with unintended pregnancies and particularly those seeking abortion. Yet, few resources exist to help health care and allied helping professionals provide culturally competent and gender-affirming abortion care to such a population group.

**Objective:**

This project aimed to redesign and adapt an existing subscription-based medication abortion mentorship platform into a culturally appropriate and gender-affirming open-access website of curated health professional resources to promote equitable, accessible, high-quality abortion care, particularly for underserved populations.

**Methods:**

We drew on a user-centered design framework to redesign the web platform in 5 iterative phases. Health care and allied helping professionals were engaged in each stage of the development process including the initial design of the platform, curation of the resources, review of the content, and evaluation of the wireframes and the end product.

**Results:**

This project resulted in an open-access bilingual (English and French) web-based platform containing comprehensive information and resources on abortion care for health care providers (physicians, nurse practitioners, and pharmacists) and allied helping professionals (midwives, medical officers, community workers, and social workers). The website incorporated information on clinical, logistical, and administrative guidance, including culturally competent and gender-affirming toolkits that could equip health care professionals with the requisite knowledge to provide abortion care for underserved populations.

**Conclusions:**

This platform contains resources that can increase the competencies of health care professionals to initiate and sustain culturally and contextually appropriate abortion care for underserved groups while clarifying myths and misconceptions that often militate against initiating abortion. Our resource also has the potential to support equitable access to high-quality abortion care, particularly for those among underserved populations who may have the greatest unmet need for abortion services yet face the greatest barriers to accessing care.

## Introduction

Access to abortion is a fundamental human right and a critical component of sexual and reproductive health care [1]. Each year, approximately 56 million abortions occur across the globe—translating to 35 abortions per 1000 women. In Canada, nearly half (~40%) of pregnancies are unplanned, and one-third of women and pregnancy-capable people will have at least 1 abortion in their lifetime [2,3]. Medication abortion using mifepristone is the international gold standard for first-trimester medication abortions and currently accounts for over 50% of all abortions in most European countries and the United States. Canada’s unique regulations allow nonphysician prescribers to prescribe and dispense the medication and allow people without access to ultrasound to access medication abortion services. Despite the loosening of restrictions in Canada, timely access to safe abortion services can be difficult for historically, persistently, or systemically marginalized (HPSM) populations including racialized groups, migrants, Indigenous people, people with disabilities, homeless and underhoused people, sex workers, two-spirit, lesbian, gay, bisexual, transgender, queer, intersex, and gender-diverse (2SLGBTQI+) people, youths, and people living in rural and remote areas [4,5]. For instance, Indigenous people in Canada experience more abortion access barriers than non-Indigenous Canadian people [6]. In addition, approximately 18% of people in Canada traveled more than 100 km to access abortion services [7]. While most clinicians in Canada indicated providing abortion services to underserved populations, the majority of them did not have the appropriate cultural training to provide care to underserved populations. For instance, a recent study suggests that most clinicians in Canada who provide abortion services to underserved populations (91.2%) reported not receiving any form of training for providing care to such populations [8]. A lack of training or mentorship in culturally safe and gender-affirming care would further exacerbate barriers to accessing abortion services that already bedevil underserved populations including Indigenous people [9,10].

To provide culturally safe and gender-affirming abortion services, there is an urgent need to develop, evaluate, and implement training and mentorship resources that will equip health care professionals in providing equitable abortion services to underserved populations in Canada. In this paper, we reported on the processes we adopted in redesigning our already-established web-based community of practice platform into an open-access website. We also highlighted on the considerations for developing a gender-affirming, culturally appropriate, and inclusive web platform to support health professionals in providing abortion to equity-deserving populations. With the internet increasingly becoming a major source of information on abortion [11], an open-access web platform could not only help in disseminating evidence-based abortion resources to all health care professionals but also help in dispelling the myths and misconceptions that often fuel abortion-related stigmas. Furthermore, an open-access web platform could assist in countering abortion-related misinformation or disinformation that is often targeted at health professionals by antichoice groups.

## Methods

### Study Design

**Figure 1.**
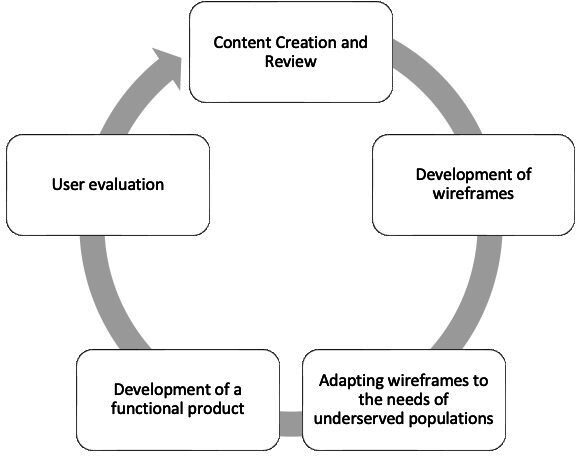
A schematic depiction of the website development process.

We used a mixed methods user-centered design approach in developing the content and structure of the website [12]. In compliance with the user-centered design process, we engaged with the relevant stakeholders in Canada (health professional associations and abortion advocacy organizations) in 5 iterative design phases. The five phases include (1) content creation and review, (2) development of wireframes, (3) adaptation of wireframes to end-user needs, (4) development of a functional product, and (5) user evaluation. We adopted a user-centered design process to emphasize the clinician-centeredness of the website while revealing the knowledge gaps that need to be addressed to create an inclusive web platform. Figure 1 shows a schematic presentation of the website’s development process.

### Ethical Considerations

This project was approved by the University of British Columbia Behavioral Research Ethics Board (approval H22-03342). All participants who took part in the interviews were provided with CAD $150 (US $103.34) honoraria.

### Phase 1: Content Creation and Review

#### Overview

The content creation phase comprised 2 steps to ensure that the content is up-to-date and reliable. These include (1) the content curation process, in which we searched for resources that are relevant to medication abortion, and (2) the content review process.

#### Content Curation

**Figure 2.**
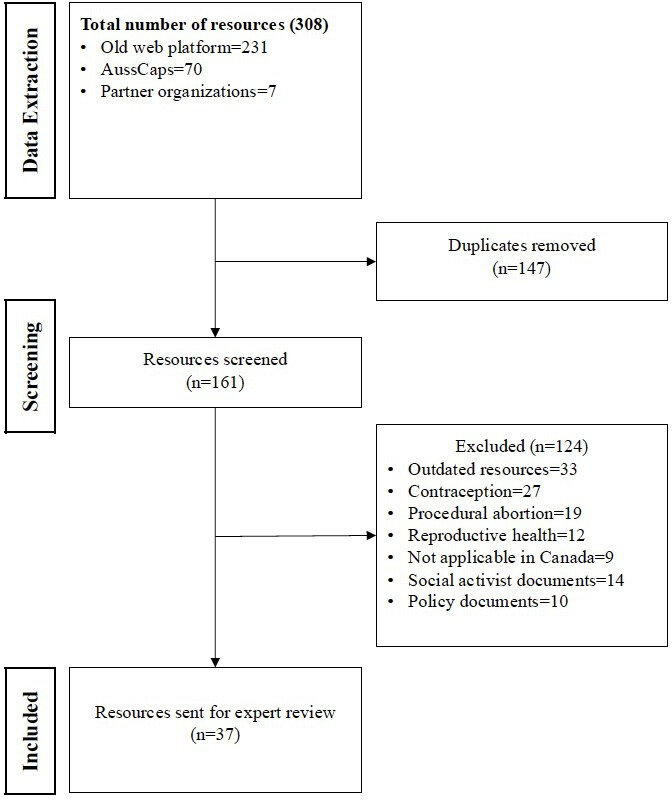
Resource extraction and screening process.

We actively engaged with health professional associations, health regulatory agencies, and sexual and reproductive health advocacy organizations across Canada to curate relevant content for the website. To ensure high-quality, appropriate, evidence-based materials specific to Canadian health care professionals, we focused our content curation efforts on sources within Canada as well as trusted and reliable international evidence on medication abortion. The main sources for curating our content include the national health professional organizations (Society of Obstetricians and Gynaecologists of Canada, Canadian Pharmacy Association, Canadian Association of Midwives, and Canadian Association of Schools of Nursing) and advocacy groups (Action Canada for Sexual Health and Rights and National Abortion Federation) as well as our already-established web-based community of practice platform [13]. We examined relevant resources from a partner website in Australia (the Australian Contraception and Abortion Primary Care Practitioner Support Network) [14]. We limited our focus to medication abortion to enhance relevance and usability for clinicians looking to adopt, improve, and sustain the practice of this newly available service in Canada. After identifying the data sources and establishing a working relationship with stakeholders, we undertook focused data extraction from May 2023 to November 2023. The data extraction process yielded a total of 308 documents detailing resources on medication abortion. These were compiled into a Microsoft Excel sheet, and 147 duplicates were removed. Following this, we conducted an initial screening of the resources by examining the titles, abstracts, headings, and subheadings to determine their relevance to medication abortion. To be eligible for inclusion, each resource must be focused on medication abortion services including clinical (guidelines, protocols, checklists, and factsheets) and nonclinical services (financial, legal, and social). Since our objective was to improve equitable access to abortion among underserved populations, medication abortion resources focusing on Indigenous populations, racialized groups, and gender minorities were included. Resources were excluded if they were dated more than 20 years ago, not relevant to the Canadian context, beyond the scope of medication abortion, or not targeted to health care providers (ie, physicians, pharmacists, social workers, and midwives). Most papers were not relevant to the Canadian context because of the constant regulatory changes regarding the prescription, dispensing, and use of medication abortion in Canada since 2017 [15-17]. We also excluded resources that were exclusively focused on procedural abortion, contraception, or general reproductive health or illnesses. Procedural abortion resources were excluded because procedural abortion demands specialized training, and we considered a web platform as an inappropriate training source for those seeking to provide this skilled task. After screening the resources against the inclusion and exclusion criteria, 124 resources were excluded, leaving us with 37 resources for a detailed expert review. Figure 2 shows the data extraction and review process.

#### Content Review

Even though the resources were extracted from credible sources, medication abortion regulations and professional practice guidelines in Canada have evolved over time, and with these changes, some of the resources might have become outdated. To ensure our website contains up-to-date resources and is in line with current practices, we engaged 10 health care and allied helping professionals providing abortion services to review the 37 resources for accuracy, currency, and relevance for providing medication abortion, particularly for equity-deserving populations. These health care professionals include physicians, nurse practitioners, pharmacists, and midwives. We asked each health care professional to review each document and respond to the following questions: (1) How do you think this resource will be relevant or useful for you to initiate or strengthen your medication abortion services and why? (2) Please indicate specific parts that you feel are most useful for your practice. (3) Please indicate specific parts that you think are out of date, not useful, or contradict your current practice.

All reviewers provided their feedback within 1 month, and all feedback was compiled into a single document. Following the review, we then engaged 2 experts in abortion care (a family physician and an obstetrician and gynecologist) to discuss and reconcile any differences or contradictory comments by the reviewers. The 2 expert reviewers resolved any differences and wrote a master document containing up-to-date resources on current medication abortion practices. The master file was organized into three main sections including (1) prescriber resources (how medication abortion works, preabortion medication evaluation, postabortion assessment, web-based and hybrid care, physician billing information, regulations, and inclusivity toolkit), (2) dispenser resources (coverage information, patient communication guidelines, checklist for dispensing abortion, and pre- and postabortion assessment), and (3) supporting resources (patient counseling before and after abortion and social worker support resources). This master document was used to inform the content of the website.

### Phase 2: Development of Wireframes

**Figure 3.**
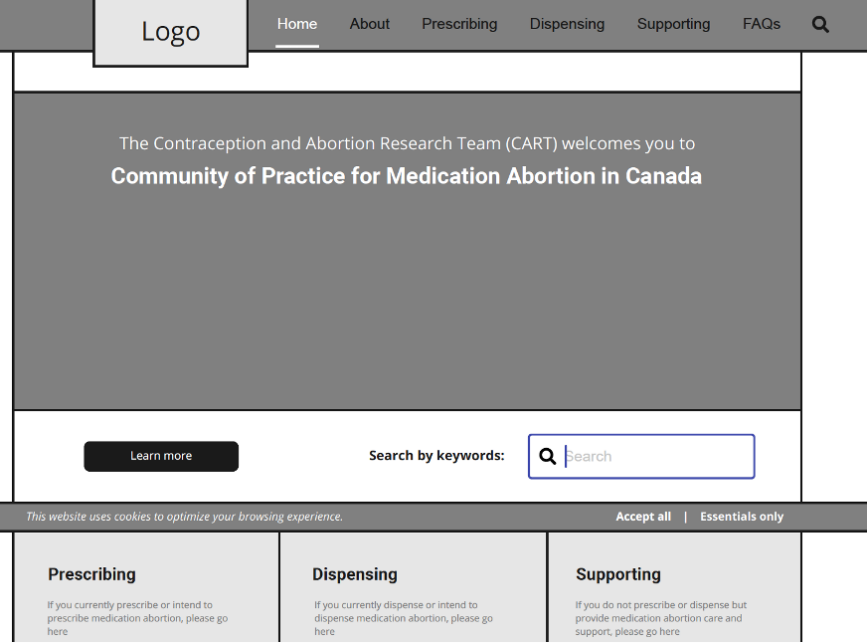
A screenshot of the wireframe.

Using a web-based design tool known as Figma (Figma Inc), we created wireframes of the website. A wireframe is a visual guide that shows a skeletal framework of a web interface [18]. The wireframe was designed to follow the format of the resources on the master file. The framework depicted the page layout and arrangement of content on the website including interface elements, navigational features, the range of functions to include, and the relative prioritization of content. This wireframe was reviewed by 3 experts in abortion care, and revisions were made regarding space allocation and content prioritization for each of the resource sections outlined in the master file. Following this review, the skeletal framework was then populated with the content from the master file. Figure 3 shows the wireframe of the website.

### Phase 3: Adapting the Wireframes to the Needs of Underserved Populations

After populating the skeletal framework with the revised content, we then conducted a focus group discussion with 14 health care professionals who currently provide, or intend to provide, medication abortion in Canada. The essence of the focus group was to understand how the wireframe can be adapted to equip health care professionals to provide inclusive, gender-affirming, and culturally safe abortions to underserved populations in Canada. We also wanted to identify potential areas for improvement before moving on to developing a functioning website.

Before the start of each focus group, we distributed a visual layout of the website containing relevant content to all participants. The participants were asked to review the visual layouts ahead of the web-based meetings. During the focus group discussions, participants were asked about (1) how the information on the wireframe could be adapted to support them in providing evidence-based, culturally safe, and accessible abortion services to HPSM populations and (2) any additional features, functionalities, and considerations for ensuring confidentiality, appropriateness, culturally affirmativeness, and supportive resources for improving access and quality of abortion seeking experience for HPSM populations.

The focus groups were led by author AFA, with assistance from RC. Each session lasted approximately 1 hour, and the data were transcribed verbatim. The data from this focus group were analyzed thematically, and the findings (reported under the Results section) were used to modify the Figma designs before moving on to developing a functional product. The findings from the focus groups helped to minimize the design flaws and reduce the possibilities for major changes once a product was eventually developed.

### Phase 4: Development of a Functional Website

**Figure 4.**
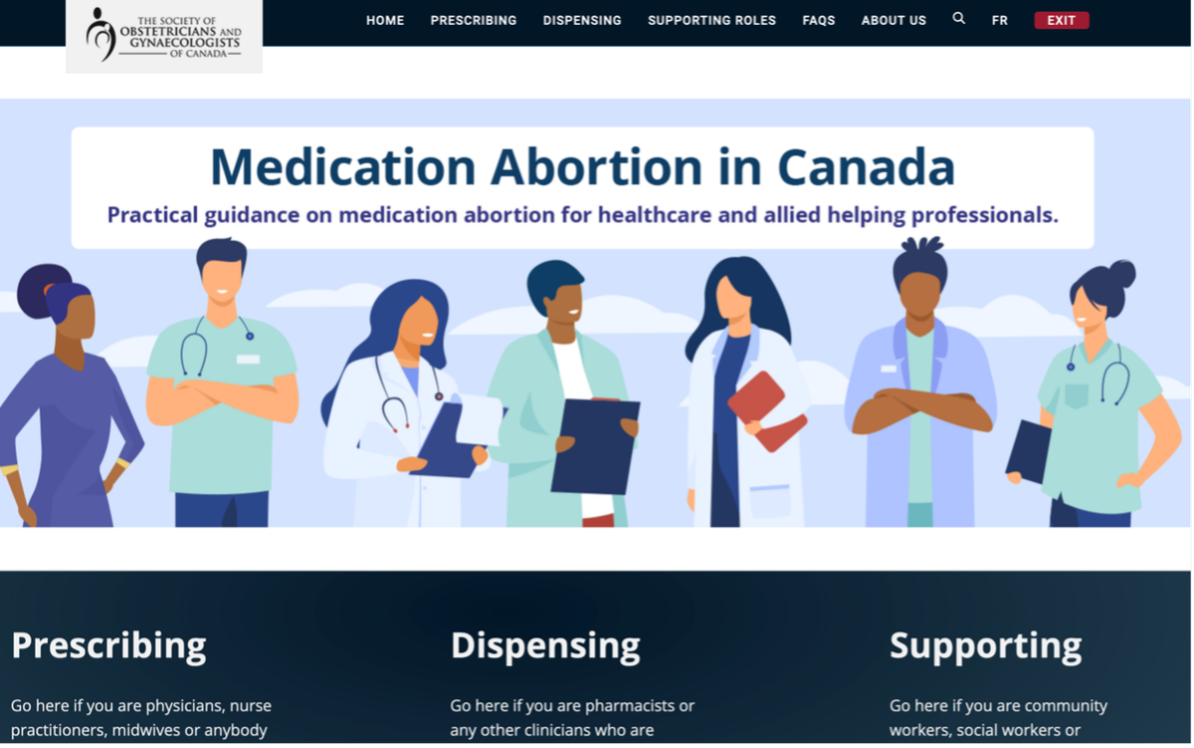
The home page of the functional website.

After making changes to the Figma design, we engaged our software engineers to translate the Figma design into a functioning website. To further explain the Figma design to the development team, we wrote a Systems Requirements Specification Document to further explain the Figma design to the development team. This document included a breakdown of the Figma designs into various components and specifying each design feature as contained in the guide [19]. Figure 4 shows the interface of the functional website. To make sure that the functional product reflects the Figma design and meets the knowledge needs of the end-user clinicians, the development team sought ongoing feedback from our team as well as from the health care professionals who reviewed the resources. We held biweekly consultation meetings with the software development team starting from August 2023 until we had a functional website in February 2024. In compliance with the user-centered design process, we evaluated the functional website with end-user clinicians.

### Phase 5: User Evaluation

We conducted a user evaluation of the website using a think-aloud observation method with 26 health care professionals across Canada. A think-aloud observation is a user evaluation method, in which participants verbalize their thought processes as they interact with a system [20]. The purpose of the user evaluation was to determine the relevance and usefulness of the website in providing abortion services to equity-deserving and HSPM populations, understand the ease of use of the website, detect any design flaws, and make final changes before the website is launched. During the think-aloud observation, we provided participants with 5 tasks related to information retrieval on our website. As participants navigated the website, they verbalized their thought processes by commenting on aspects of the website that they found confusing, difficult to navigate, or irrelevant to their purpose. The think-aloud data were analyzed using simple content analysis. The findings (reported under the Results section) were used to revise the website.

## Results

### Overview

In this section, we focus on the findings from the focus group discussions of the wireframes as well as the user evaluation of the functional website. Overall, the health professionals who took part in the focus group discussions and the end-user evaluation acknowledged that our web platform was an important educational resource that would equip them with the necessary knowledge to provide abortion care. They also provided recommendations on how this web-based platform could be adapted to support them in providing inclusive abortion care to underserved populations.

### Findings From the Focus Group Discussion

#### Overview

The participants expressed their satisfaction with the layout and the content displayed on the wireframes. They indicated that the various clinical guidelines, factsheets, and checklists on medication abortion would help demystify the myths and misconceptions associated with providing abortion. The usefulness of the resources in demystifying abortion provision was a very important feature considering participants’ self-described limited understanding of medication abortion. While acknowledging the importance and potential usefulness of the wireframes, participants noted some limitations on the interfaces of the wireframes and suggested ways in which they could be adapted to improve the capacity of health care professionals to provide abortion services to equity-deserving and HPSM populations. These recommendations fell into 4 thematic areas.

#### Inclusive and Culturally Sensitive Content

Participants expressed the need for the website to contain inclusive and culturally sensitive language that has the least chance of causing harm to underserved populations including Indigenous, trans, and nonbinary people who seek abortion. To enable health professionals to provide inclusive abortion services to trans people, the participants suggested that the website should contain gender-neutral terms as well as handouts and factsheets on abortion and postabortion care in multiple languages. A physician who provides abortion stated:

A lot of my patients can speak English but there are instances [where] I get patients that cannot speak English properly. In such cases, it will be good to have some resources in their language that I can give out to them.

Some of them also suggested having a print-out function to provide materials for patients who do not speak English. The participants also expressed the need for low bandwidth connectivity to enable health care providers in rural areas with poor internet access to access resources.

#### Indigenous and Gender-Diverse Resources

The participants also called for the inclusion of Indigenous and 2SLGBTQI+ resources on the website because they indicated that one of the groups facing the most barriers to accessing health services includes Indigenous groups and the 2SLGBTQI+ populations. This could be seen in one of the statements given by one of the participants:

it isn’t just about understanding the clinical guidelines and factsheets. But we also need to understand the other aspects of Indigenous cultural safety when providing abortion to Indigenous patients like the relationship between abortion care and legacies of forced apprehension/the millennial scoop, information on the knowledge and risk of reproductive coercion (either for or against abortion) etc.

These calls were particularly made by participants who provide services to Indigenous communities. Other participants indicated a need for a learning hub or short courses on Indigenous cultural safety, gender-affirming, and trauma-informed abortion care—arguing that these additional knowledge sources will help them to provide services to abortion seekers who experienced historical and intergenerational traumas.

#### Providing Resources for Noninsured Clients

The participants also indicated how the website could be a “one-stop shop” of information if they included information on how to support noninsured clients. This recommendation was made by a midwife who revealed the challenges of providing abortion services for noninsured clients such as undocumented migrants:

More than half of my clients were unhoused, or street-involved or living in extreme poverty, or they were refugees, recently landed immigrants, or people of color and trans communities. You know there is a significant intersection between such populations and those living in poverty so there needs to be some resources around how to make sure that these populations are also getting access to abortion services.

#### Protecting the Privacy of Website Users and Abortion Seekers

The participants acknowledged the stigmatizing nature of abortion and the need to protect people’s privacy. Thus, they suggested including a built-in exit button that would enable users to immediately exit the website, particularly in public places. They also indicated the need for the website to contain geolocation features that can map out nearby abortion providers and pharmacies outside of people’s communities. One participant stated:

I provide services in a local community, and some don’t feel comfortable seeking abortion services from me because I may probably know them. When this happens, it’s difficult to refer because they won’t even come to you. So, it will be good to have a feature on this platform where patients themselves can search for nearby abortion providers that they can approach with comfort.

According to the participants, this will make visible nearby abortion providers that could serve as alternative sources for patients who may be reluctant to seek abortion services within their (particularly very small) communities due to privacy concerns.

### Findings From the User Evaluation

#### Overview

Iterative feedback from the focus group informed changes to the website, as participants serially identified gaps and problems to be addressed before it was launched. Some health care professionals indicated that the functional website felt too medical and was insufficiently focused on the social aspects of abortion. They perceived our banner image on the home page as overrepresenting physicians and not sufficiently acknowledging the role of midwives and supporting professionals. They also made recommendations on how the website could be adapted for navigability and to touch on the stories of underserved populations. The recommendations from the user evaluation fell into 3 thematic areas.

#### Portraying a Positive Image of Abortion via Visual Effects

Some health care professionals commented that the initial wireframes lacked a human touch and thus suggested adding more vibrant and bright colors that reflect the identities of diverse groups of health professionals and the diversity of patients. Participants felt that such adaptations could portray abortion as something that is considered normal for everyone to seek, normal for every health professional to provide or support, and to ensure the abortion-seeking experience is positive.

maybe some colors will be nice because I feel that when I am looking into abortion care, I just want to take this stigma away. So, having colors that are dark for me is like you are doing something bad, right? So maybe having some lighter colors and highlighting on socio-cultural aspects of abortion would be helpful. People need to feel that abortion is normal after going through this website.

#### Connecting With Patients

Other health professionals explained that although the website targets health care professionals, it is ultimately about caring for people. They thus suggested that it would be great to have images of health care providers taking care of patients from diverse gender and racial backgrounds or better still include images of patients having children to remove the myth that people seeking abortion are irresponsible adults without children or families. One participant stated:

This website is about people getting care. So, we need to see how so many people who have pregnancy termination also have children already. So, it’s not just like the unwed single woman who wants to terminate her pregnancy.

The participants also expressed the need for the website to help health care professionals understand the patient populations most likely to seek abortion services from them. This way, the website would not only provide clinical information but also help providers to connect with the patients’ stories.

#### Making Compromises Between Being Comprehensive and Practical

Many participants acknowledged the comprehensiveness of the website and were confident that they would be able to find relevant information should the need arise. They also acknowledged that being comprehensive might make the website a less suitable quick reference guide, particularly during clinician-patient encounters. This concern could be a challenge for high-volume providers and providers in rural and remote areas. One midwife stated:

the information that I’m seeing is actually really good. It’s really good information, and I’m intrigued to read more about what’s in here. But [it’s] too much. It would be really good for like education and training, but not when you have a patient in front of you.

While this theme was not specifically related to providing abortion to underserved populations, it highlighted the busy schedule of clinicians vis-à-vis looking for information in a dense and cluttered web platform.

## Discussion

### Principal Findings

In this study, we report on the cocreation of an open-access web-based abortion platform and highlight considerations for promoting access to abortion for underserved populations. This study followed an intersectionality study we conducted with end-user clinicians before embarking on the development of the website [21]. Our study incorporated rigorous engagement of end-user clinicians, and our user evaluation data demonstrated an accessible, acceptable website that could better equip health and allied helping professionals to provide equitable, culturally safe, and gender-affirming resources to equity-deserving and HSPM populations. It is important to note that many health professionals in Canada, including nurse practitioners and midwives, had not received any formal training on abortion as part of their education, leading to limited knowledge and general uncertainties in providing abortion [22]. The limited knowledge may lead to some hesitation in providing abortion, particularly for patients from underserved populations who present with complex health challenges. We believe that this mentorship platform will equip health professionals with the necessary knowledge to provide abortion to patients who face intersecting barriers to access services while also enhancing timely decision-making for health professionals in rural and remote areas. For a time-sensitive procedure such as abortion, expeditious decision-making is crucial to high-quality care for women and pregnancy-capable people who request abortion services.

In addition to information dissemination and helping with decision-making, an open-access abortion web platform may also serve to counter abortion-related misinformation and disinformation that are often targeted at health care professionals. This is important at a time when the proliferation of disinformation, together with the stigma and belief-based refusal to provide legal and appropriate abortion care, creates interpersonal tensions that make it difficult for some health professionals to meet the reproductive needs of their patients [23,24]. Health professionals may further be disadvantaged when there are frequent changes in medication abortion guidelines and regulations [17]. These changes, together with the need for quality improvement, highlight the importance of such a web-based platform for updating the competencies of health care professionals who provide or intend to provide abortion. This is more relevant, as there is an increasing call for the timely integration of research evidence in routine clinical practice [25].

Our findings suggest that adopting a user-centered design in this project resulted in a platform that was largely considered by potential end-user clinicians as inclusive. While engaging stakeholders from diverse clinicians and organizations was quite challenging, we acknowledged that the process was essential to fulfill the principles of user-centered design. Our team believes that the active engagement of clinicians, health professional organizations, and abortion advocacy groups in cocreating this web-based platform enhanced the appropriateness of the content while facilitating clinician recruitment, dissemination, and subsequent uptake of the resource among end-user health care professionals.

It is also important to note that few digital health projects are designed from gender equity and Indigenous rights perspective [26]. In the context of abortion-related websites, intersectionality is particularly important, as it helps illuminate ways in which the website could be tailored to the needs of the population subgroups facing the most barriers in access to abortion (racialized groups, migrants, people with disabilities, homeless and underhoused people, sex workers, 2SLGBTQI+ people, Indigenous people, and youths) [21,27]. Indeed, available evidence suggests that such minority groups are overrepresented among those with unintended pregnancies and particularly among those seeking abortion [28-30]. Therefore, engaging with a group of health professionals who serve diverse populations in developing and evaluating our web platform made the website unique, as it cedes all decisions on the content and structure in the hands of the end-user clinicians who may have first-hand experience in providing services to underserved populations. This approach to developing our website supports the vision of “design justice” where the community owns the design artifacts [31]. By cocreating with health care professionals, we were able to generate website content that illuminates ways health care professionals can provide abortion services to underserved populations who present with complex, historical, and intersecting health challenges. This approach is more likely to lead to a seamless translation of the web platform into practice since the clinicians and partner organizations were more or less coowners of the end product.

Creating web platforms that are inclusive of equity-deserving and HSPM populations requires more than just technically sophisticated algorithms but a deep understanding of users’ needs and specific considerations that promote inequities in access to health care [32]. The inclusive design recommendations from the participants in this study were aimed at equipping health care professionals with the necessary tools to address inequities in access to abortion care. The integration of low-bandwidth connectivity for instance would improve accessibility of the resources to health professionals in rural and remote areas with low internet connectivity. Improved web access could help improve abortion access in rural and remote areas where medication abortion might be the only, or most accessible, option [33]. Furthermore, the recommendation for including geolocation services could help address abortion-related stigma and provide better privacy for abortion seekers in communities with conservative values around abortion. While the argument for geolocation features was seen as a way of helping patients easily locate abortion providers and pharmacists, some were concerned that geolocation services could be used by antichoice groups to target abortion providers. Additional security measures may be needed if geolocation features are to be implemented on abortion-related web platforms.

### Limitations

Our study was not without limitations. Even though our objective was to design a web platform that supports health care professionals to provide equitable access to abortion for underserved populations, we did not rigorously engage abortion seekers the same way we engaged with clinicians. A future improvement would be to engage the patients’ voices in resource development to better reflect the peculiar needs of underserved populations. Our ultimate goal for this website is to empower health care professionals to provide equitable and inclusive abortion to underserved populations. However, we cannot ascertain if we have achieved that objective at the time of writing this paper. Future evaluation reports from users would tell us if the resources provided in this platform are indeed useful in providing abortion services to underserved populations. Furthermore, the participants made several recommendations in the focus group and the user evaluation that were not implemented mainly due to time and resource constraints. While we acknowledge this as a limitation, it is not uncommon to see such limitations in digital health projects, as it is practically impossible to implement all findings from user evaluation [34,35]. Finally, even though we planned to recruit health professionals who identify as or have expertise in providing abortion services to underserved populations (ie, racialized people, migrants, people with disabilities, homeless and underhoused people, sex workers, 2SLGBTQI+ people, and youths as well as those living in or providing abortion services in rural or remote areas), it was difficult to find health professionals who identify as or has expertise in all these diverse populations. Therefore, some phases were completed without adequate input from health professionals with expertise or experience in providing abortion services to underserved populations.

### Conclusions

Drawing on a user-centered design approach, we cocreated a gender-affirming and culturally appropriate open-access mentorship platform with and for health care professionals to support the equitable provision of abortion in Canada. We believe that a platform of this nature would increase medication abortion awareness among health care professionals and destigmatize abortion care while clarifying the myths and misconceptions that often create tensions and foment general uncertainties in initiating and sustaining abortion care. With underserved populations more likely to have unintended pregnancies than the general population [36,37], a website that specifically seeks to improve the competencies of health care professionals in providing abortion services to this population is important and timely. We believe that this platform has the potential to enhance the knowledge and expertise of health care professionals, particularly those in rural and remote areas on how to initiate and sustain abortion care for HPSM populations in Canada who face the most barriers in access to health services. This platform is also expected to provide the necessary resources needed to support health care professionals in providing evidence-based and culturally safe medication abortion for health care professionals in rural and remote areas.

## Abbreviations

2SLGBTQI+: two-spirit, lesbian, gay, bisexual, transgender, queer, intersex, and gender-diverse
HPSM: historically, persistently, or systemically marginalized
